# Targeted Facebook Advertising is a Novel and Effective Method of Recruiting Participants into a Human Papillomavirus Vaccine Effectiveness Study

**DOI:** 10.2196/resprot.5679

**Published:** 2016-07-22

**Authors:** Asvini K Subasinghe, Margaret Nguyen, John D Wark, Sepehr N Tabrizi, Suzanne M Garland

**Affiliations:** ^1^ Royal Women's Hospital Department of Microbiology and Infectious Diseases Parkville Australia; ^2^ Murdoch Childrens Research Institute Infection and Immunity Theme Parkville Australia; ^3^ Royal Melbourne Hospital Department of Medicine University of Melbourne Parkville Australia; ^4^ Royal Melbourne Hospital Department of Bone and Mineral Medicine University of Melbourne Parkville Australia; ^5^ University of Melbourne Department of Obstetrics and Gynaecology Parkville Australia

**Keywords:** online recruitment, social media, Facebook, human papillomavirus, HPV

## Abstract

**Background:**

Targeted advertising using social networking sites (SNS) as a recruitment strategy in health research is in its infancy.

**Objective:**

The aim of this study was to determine the feasibility of targeted Facebook advertisements to increase recruitment of unvaccinated women into a human papillomavirus (HPV) vaccine effectiveness study.

**Methods:**

Between September 2011 and November 2013, females aged 18 to 25 years, residing in Victoria, Australia, were recruited through Facebook advertisements relating to general women’s health. From November 2013 to June 2015, targeted advertising campaigns were implemented to specifically recruit women who had not received the HPV vaccine. Consenting participants were invited to complete an online questionnaire and those who had ever had sexual intercourse were asked to provide a self-collected vaginal swab. The HPV vaccination status of participants was confirmed from the National HPV Vaccination Program Register (NHVPR).

**Results:**

The campaign comprised 10 advertisements shown between September 2011 and June 2015 which generated 55,381,637 impressions, yielding 23,714 clicks, at an overall cost of AUD $22,078.85. A total of 919 participants were recruited. A greater proportion of unvaccinated women (50.4%, 131/260) were recruited into the study following targeted advertising, compared with those recruited (19.3%, 127/659) prior to showing the modified advertisement (*P*<.001). A greater proportion of the total sample completed tertiary education and resided in inner regional Victoria, compared with National population census data (*P*<.001), but was otherwise representative of the general population.

**Conclusions:**

Targeted Facebook advertising is a rapid and cost-effective way of recruiting young unvaccinated women into a HPV vaccine effectiveness study.

## Introduction

Human papillomavirus (HPV) is the most common sexually transmitted viral infection worldwide. Approximately 80% of sexually active individuals will acquire an HPV infection during their lifetime; most within a few years following sexual debut [1]. Persistent infection with high-risk or oncogenic HPV genotypes, particularly HPV 16 or 18 is a prerequisite to cervical cancer [2].

In 2007, Australia was the first country to implement a national government-funded HPV vaccination program using the quadrivalent HPV (6, 11, 16, and 18) vaccine Gardasil (4vHPV) [3-6]. The vaccine is available free-of-charge to 12 to 13 year old girls as an ongoing program and had a catch-up component for up to 26 year olds to December 2009, with vaccinations administered through schools, general practices and other community health services. Since the initiation of the program, the National HPV Vaccination Program Register (NHVPR) has documented high vaccine uptake, with coverage rates of approximately 85% of females aged 15 having received at least 1 dose, and 77% having completed the 3 dose course in 2015 [7].

Although Internet social networking sites (SNS) have been in use for over a decade, the concept of targeted advertising as a recruitment strategy in health research is still in its infancy. Approximately 93% of Australians use Facebook, including 97% of 18 to 29 year olds [8]. Of those aged 18 to 29, 79% of females use Facebook at least daily [8]. The increasing pervasiveness and utility of social media as powerful communication channels means that SNS, such as Facebook, can potentially be used to effectively engage young people in health studies. There is evidence to show that Internet-based research can yield high response rates at a considerably lesser cost than that accrued by traditional recruitment methods [9-12]. In addition, findings from pilot studies demonstrate that Facebook advertising is a feasible recruitment strategy for health studies and yields a broadly representative sample of a target population [10,13-19]. In one particular study, investigators looked at geographic variation in HPV vaccine uptake in men and women using targeted Facebook advertisements to recruit residents in Minnesota [18]. However, this has only just been utilized as a methodology in Australia in the Vaccine Against Cervical Cancer Impact and Effectiveness (VACCINE) study [20].

The VACCINE study is a cross-sectional survey in which Facebook was used to recruit participants [21]. The objective of this study was to investigate the changes in prevalence of vaccine-targeted HPV genotypes in a cohort of 4vHPV vaccine-eligible young women aged 18 to 25 years living in Victoria, Australia. Within the recruitment strategy we also modified the initial Facebook advertising campaign to specifically target and over-recruit unvaccinated women, to better understand why a free vaccine was not being embraced. We hypothesized that targeted advertising through Facebook enables faster and more efficient recruitment of vaccine-eligible women who have not yet received the HPV vaccine compared with non-targeted advertising.

## Methods

The methods for this study have been published previously [21]. The study protocol was approved by the Human Research and Ethics Committee at the Royal Women’s Hospital, Melbourne, Australia.

### Participant Recruitment and Inclusion Criteria

Participants were recruited through advertisements published on Facebook. From September 2011 to November 2013, advertisements were set to randomly appear to Facebook users who were (1) female; (2) between the ages of 18 and 25 years; and (3) residing in Victoria, Australia.

**Figure 1:**
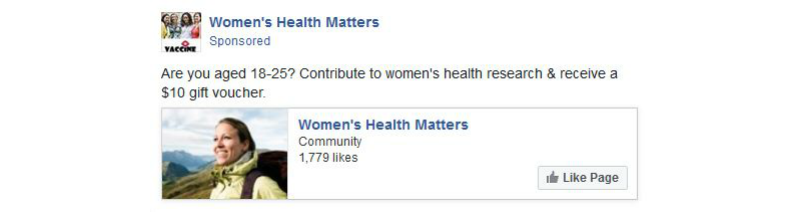
Example of an original advertisement from the VACCINE Study’s Facebook advertising campaign.

**Figure 2:**
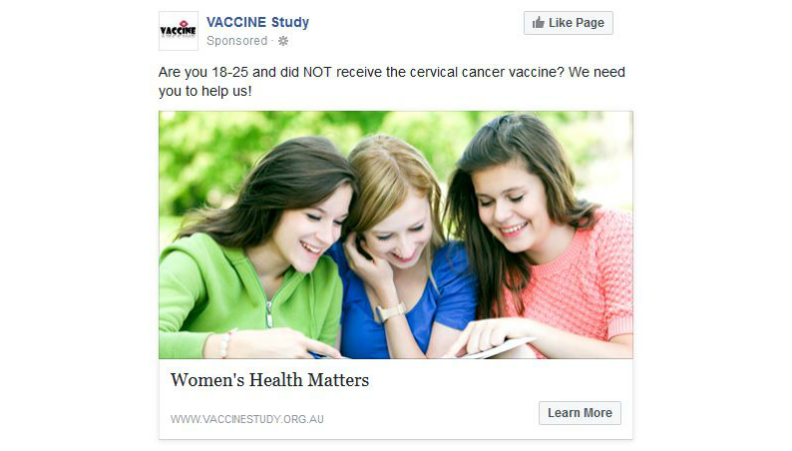
Example of a modified advertisement from the VACCINE Study’s Facebook advertising campaign.

The advertisements contained a brief headline (eg, “Women’s Health Matters”), a generic picture of young women and a brief caption (Figure 1). The advertisements were subsequently modified by changing the text to target women who had not received the 4vHPV vaccine, and made visible to Facebook users from November 22, 2013 to June 2015 (Figure 2). The decision to modify these advertisements arose mid-way through the study as we wished to understand the determinants of women eligible for, but not accepting, the HPV vaccine.

Respondents could click through to the secure VACCINE Study website to read more about the study and register an expression of interest (EOI). Potential participants were contacted and screened by telephone to assess their eligibility and willingness to comply with the study requirements.

### Measures

Participants who verbally consented to the study were invited to provide electronic consent and to complete a self-administered, password-protected questionnaire hosted by the online survey tool SurveyMonkey. The survey questions related to demographic characteristics, sexual history and knowledge, attitudes and practices regarding HPV, the HPV vaccine, and cervical cancer screening. Participants were also requested to provide their HPV vaccination status and written or electronic consent for their HPV vaccination history to be verified with the NHVPR.

### Statistical Analyses

Descriptive statistical analyses were conducted using Stata 13.1 (StatCorp LP, College Station, TX, USA). The demographics of our cohort were compared with general population data sourced from the Australian Bureau of Statistics 2011 census data [22]. Socioeconomic status was assigned using the Postal Area of Relative Socio-Economic Disadvantage 2011 [22]. The Chi-square test was used for all demographic comparisons between our sample of study and the general population, as well as within study comparisons between females recruited by general advertisements and those recruited by targeted advertisements. A 2-sided *P* value <.05 was considered statistically significant. Data were treated as missing if a question was skipped or “prefer not to answer” was selected.

Participants who received all 3 doses of the HPV vaccine were considered “vaccinated”; those who had received 1 or 2 doses were considered “under-vaccinated” and women who never received the HPV vaccine were recorded as ”unvaccinated“.

## Results

For the duration of the entire campaign (September 2011 to June 2015), 10 advertisements resulted in 55,381,637 impressions, reaching 984,159 people, and yielding 23,714 clicks, at an overall cost of AUD $22,078.85. This translated to an average cost of AUD $24.02 per participant. The general advertisements which were implemented from September 2011 to November 2013, made 35,906,205 impressions, yielding 15,304 clicks, with an overall cost of AUD $15,381,92. The average cost per click was AUD $1.01 with a click-through rate of 0.04% per impression. From November 2013 to June 2015, 19,475,432 impressions were made, yielding 8410 clicks, with an overall cost of AUD $6696.93. The average cost per click was AUD $0.80 .

**Figure 3:**
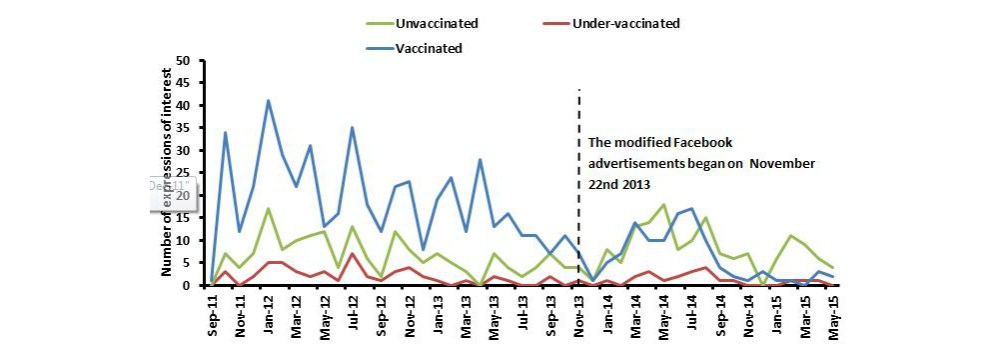
Participant recruitment rate based on date of expression of interest (EOI) (N=919).

A total of 919 participants completed the online questionnaire. Among women who were recruited and completed the questionnaire following modification of the advertisements, 50.1% (131/260) were unvaccinated. In contrast, only 19.3% (127/659) of the participants who completed the questionnaire prior to modification of the advertisements had never been vaccinated (*P*<.001) (Figure 3). There were no significant differences in socio-demographics of the unvaccinated group recruited prior to targeted advertisements with those recruited post targeting (data not shown).

No significant differences were detected between the general population participating in the VACCINE study to those that were then targeted for not being vaccinated against HPV, except that for the latter group a greater proportion were born outside of Australia (19.1% vs 12.4%, *P*<.001) (Table 1).

**Table 1:** Demographic characteristics of participants recruited by non-targeted advertisements compared with those recruited by targeted advertisements in the VACCINE study (N=919).

Characteristic		Non targeted advertisement (n=659), n^a^ (%)	Targeted advertisement (n=260), n^a^ (%)	*P* value^b^
Age (years), median (Q1-Q3^c^)		22 (20-23)	22 (20-24)	.08
Geographic region				
	Major city	510 (77.3)	212 (81.5)	.5
	Inner regional	127 (19.3)	40 (15.4)	
	Outer regional/remote	21 (3.3)	8 (3.1)	
Country of birth				
	Australia	574 (87.6)	208 (80.9)	<.001
	Other	81 (12.4)	52 (19.1)	
Indigenous status				
	Aboriginal or Torres Strait Islander	5 (0.8)	3 (1.2)	.6
	Other	654 (99.2)	257 (98.8)	
Socioeconomic level (SEIFA decile)^d^				
	1-5	214 (32.6)	75 (29.2)	.3
	6-10	443 (67.4)	182 (70.8)	
Highest level of education completed^e^				
	< Year 12	29 (4.4)	14 (5.4)	.7
	Year 12	257 (39.0)	98 (37.7)	
	> Year 12	365 (55.4)	145 (55.8)	
Relationship status				
	Single	237 (36.0)	91 (35.0)	.7
	Casual relationship	60 (9.1)	30 (11.5)	
	Committed relationship	350 (53.1)	136 (52.3)	

^a^Numbers may not add up to the total due to missing data.

^b^Chi-square test was used for all demographic comparisons.

^c^Q1: 25th percentile; Q3: 75th percentile.

^d^Based on postal area code. Deciles are rankings within Victoria, Australia. The lowest 10% of areas are assigned a decile number of 1 and the highest 10% of areas are given a decile number of 10. Decile 1 is the most disadvantaged relative to the other deciles.

^e^Year 12 is the final year of high school in the Australian education system.

The age distribution of participants reflected the general population, with the median age being 22 years (Q1 25th percentile to Q3 75th percentile: 20-23) (Table 2). Compared with the 2011 census data, more women who enrolled in this study were born in Australia (86 % vs 76%, *P*<.001) and had completed tertiary education (57% vs 43%, *P*<.001). Women living in inner regional areas were over-represented (18% vs 16% of the total, *P*<.001) (Table 2).

**Table 2:** Demographic characteristics of participants in the VACCINE study compared with the general population in Victoria, Australia (N=919).

Demographic		Total study population^a^, n (%)	Target population^b^, %	*P* value^c^
Age group, years				
	18-21	306 (33.3)	48.6	<0.001
	22-25	613 (66.7)	51.4	
Geographic region				
	Major city	722 (78.6)	80.7	0.1
	Inner regional	167 (18.2)	16.3	0.001
	Outer regional/remote	30 (3.3)	3.1	0.6
Country of birth				
	Australia	782 (85.8)	75.5	<0.001
	Other	130 (14.2)	24.5	
Indigenous status				
	Aboriginal or Torres Strait Islander	8 (0.9)	0.9	1.000
	Other	905 (99.1)	99.1	
Education level^d^				
	Completed year 12 or below	398 (43.4)	57.4	<0.001
	Completed tertiary education	519 (56.6)	42.6	

^a^Numbers may not add up to 919 due to missing data.

^b^Population data were sourced from the 2011 Australian Bureau of Statistics Census, with figures corrected for non-responses to add up to 100%.

^c^The Chi-square test was used for all demographic comparisons.

^d^Year 12 is the final year of high school in the Australian education system.

## Discussion

### Principal Findings

Targeted Facebook advertising led to increased recruitment of young women who had not received the HPV vaccine, without employing other recruitment methods. The rationale behind over-recruiting unvaccinated women was to allow us to reliably measure any difference in the prevalence of high-risk HPV between unvaccinated and vaccinated women. We also show that recruiting through Facebook is cost-effective given that the cost per participant in this study was AUD $24.02.

Evidence from previous studies has shown that targeted Facebook advertising is effective in recruiting participants into health research [10,23,24]. In these studies, recruitment was targeted based on broad demographic characteristics such as gender, age, and location, to maximize generalizability [10,23,24]. However, there have been few studies in which Facebook advertising has been used to recruit participants with more specific characteristics [25,26]. For example, young adults who were cigarette users were sought in a study of tobacco and substance use [9,27].To attract their target audience, investigators developed Facebook advertisements which were shown to users whose profile pages contained tobacco- or marijuana-related keywords drawn from their listed interests, activities, job titles as well as the Facebook pages they ”liked“ or groups to which they belonged [9]. Our study differed slightly from this approach. Instead of using keywords to define the people to whom our advertisements were displayed, we relied primarily on Facebook users to read and respond to the text in our customised advertisements.

Our study sample compared well with the general population in age. The significantly greater proportion of women born outside of Australia recruited via targeted advertisements compared with those recruited through general advertisements is intuitive as overseas students are not eligible for the HPV vaccine. Although young women who were born in Australia and/or had completed tertiary education were over-represented in the total sample; these biases are common in population-based studies [10,24,28]. This is perhaps because highly-educated people are more likely to be aware of health issues. Therefore, highly-educated people may choose to participate in health research to address their personal health concerns and/or because of altruistic motives such as the desire to contribute to medical knowledge and improving the health of others [29]. The difference in the distribution of women living in inner regional areas in our study sample was statistically significant but small compared with the general population (2%, *P*<.001); a larger sample size is required to determine whether this is meaningful.

There is an inherent risk of introducing sampling bias when targeting Facebook advertising for specific characteristics. We found that our sample was reasonably representative of the general population, except for country of birth and education level. Another potential cause of bias associated with this recruitment method is snowball or chain-referral sampling, whereby users exposed to these advertisements share information about the study on their Facebook profile page with their friends, or relatives, who may then submit an EOI. We found that 78% heard about the study from the Facebook advertisements, whereas approximately 17% either read a post on their friend’s Facebook wall or were told by a friend to participate. These 17% referrals were constant pre and post changes to the advertisements. Given the small proportion who submitted an EOI without having seen the Facebook advertisements, we contend that this did not have a significant impact on our results.

### Conclusion

We have demonstrated the utility of paid, targeted Facebook advertising as a contemporary and effective recruitment method. The ability to specifically target individuals with particular characteristics by tailoring Facebook advertisements enables researchers to recruit specific groups of individuals of interest into health studies.

## Abbreviations

4vHPV: Quadrivalent human papillomavirus vaccine
EOI: expression of interest
HPV: human papillomavirus
NHVPR: National human papillomavirus vaccination program register
SNS: social networking sites
VACCINE: Vaccine against cervical cancer impact and effectiveness
